# *In Vivo* Confocal Microscopy as a Prognostic Indicator in *Acanthamoeba* Keratitis: Insights from a Retrospective Study

**DOI:** 10.3390/pathogens15030262

**Published:** 2026-03-02

**Authors:** Yiping Han, Yuan Wei, Qiankun Chen, Jinding Pang, Qingquan Shi, Qingfeng Liang

**Affiliations:** Beijing Institute of Ophthalmology, Beijing Tongren Eye Center, Beijing Tongren Hospital, Capital Medical University, Beijing 100005, China; sduhyp61@163.com (Y.H.); doctorweiyuan2022@163.com (Y.W.); qiankunchen1995@163.com (Q.C.); jindingpang@gmail.com (J.P.); qingquanshi2022@sina.com (Q.S.)

**Keywords:** *Acanthamoeba* keratitis, *in vivo* confocal microscopy, morphological features, prognostic model

## Abstract

Background: To assess longitudinal changes in the *in vivo* confocal microscopy (IVCM) features during *Acanthamoeba* keratitis (AK) treatment and develop a prognostic model. Methods: This retrospective study included 59 AK patients who underwent IVCM at baseline and 1 and 3 months. Fourteen morphological features covering pathogen-related characteristics, cyst arrangement patterns, and inflammatory markers were compared between good and poor prognosis groups, which were defined based on clinical outcomes including corneal perforation, the need for therapeutic keratoplasty, or final best-corrected visual acuity (BCVA) ≤ 0.05. Prognostic modeling was performed exclusively using baseline IVCM features and applied univariable and Firth-corrected multivariable logistic regression with collinearity assessment and clinical filtering, followed by 5-fold cross-validation. Results: Among 59 AK patients, 45 (76.3%) had a good prognosis and 14 (23.7%) had a poor prognosis. Poor prognosis eyes showed a higher prevalence of double-walled cysts, trophozoites, and clustered cysts, along with higher cyst density and deeper stromal invasion. In contrast, good-prognosis eyes had more target-like cysts, immature dendritic cells, and mature dendritic cells. Clustered cysts independently predicted poor prognosis (OR = 2.98), whereas target-like cysts (OR = 0.26) and mature dendritic cells (OR = 0.37) were protective (AUC = 0.883; all *p* < 0.05). Conclusions: IVCM provides a quantitative tool for early outcome prediction and individualized management. Higher cyst burden, clustered cysts, and persistent stromal involvement indicated poorer prognosis, whereas target-like cysts and mature dendritic cells indicated better prognosis.

## 1. Introduction

*Acanthamoeba* keratitis (AK) is an uncommon yet sight-threatening corneal infection with a rising global incidence [1,2,3,4]. It accounts for approximately 2% of infectious keratitis cases worldwide [2]. Recent estimates suggest a prevalence of 2.9 cases per million population, equivalent to roughly 23,500 new cases annually [3]. Its early signs often resemble viral keratitis, contributing to frequent misdiagnosis and delayed treatment, which can result in ulceration, perforation, and even the need for corneal transplantation [4,5,6]. Conventional diagnostic methods, including corneal scraping and culture, are time-consuming and may delay timely clinical decision-making [7,8,9]. *In vivo* confocal microscopy (IVCM) offers a noninvasive, real-time diagnostic solution, providing high-resolution images that identify cysts and inflammatory changes at the cellular level [3,10,11,12,13,14,15,16].

Current IVCM studies on AK have primarily focused on diagnostic applications, particularly the identification of characteristic pathogen morphologies, such as double-walled cysts, and differentiation from other infectious keratitides [11,12,13,14]. To our knowledge, no prior study has systematically integrated longitudinal IVCM features into a multivariable prognostic model for AK [11,14,15,17,18]. Accordingly, integrating pathogen morphology, inflammatory response, and structural distribution into outcome-based risk stratification is crucial for refining prognostic evaluation and optimizing risk stratification in patients with AK [13,14,18,19].

This study evaluates the longitudinal evolution of IVCM features in patients with AK and examines their independent prognostic value for clinical outcomes. By integrating multi-timepoint imaging data with outcome-based modeling, we aim to facilitate early risk stratification and support individualized management strategies.

## 2. Materials and Methods

### 2.1. Study Design

**Figure 1:**
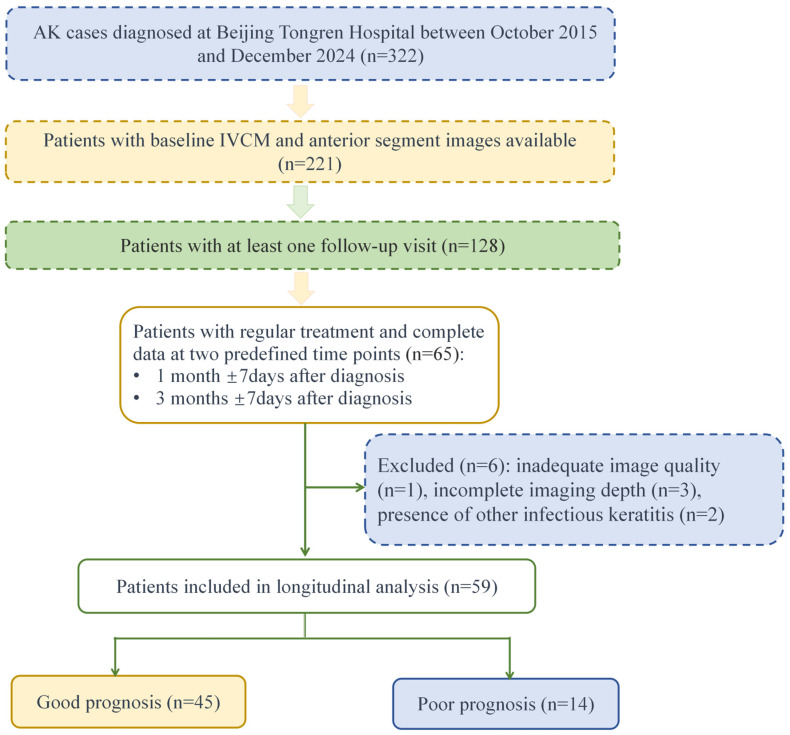
Flowchart of patient selection for IVCM analysis in *Acanthamoeba* keratitis. A total of 322 AK patients were initially diagnosed at our institution between October 2015 and December 2024. After excluding cases without baseline IVCM or anterior segment imaging, those lost to follow-up, cases with inadequate image quality or insufficient imaging depth, and cases with other forms of infectious keratitis, 59 patients with complete imaging data at baseline, 1 month (±7 days), and 3 months (±7 days) after diagnosis were included in the longitudinal analysis. Two patients presented with bilateral AK. To ensure statistical independence, only one eye per patient (the first affected eye) was included in the final statistical analysis, resulting in 59 patients (59 eyes). These were further categorized into good prognosis (n = 45) and poor prognosis (n = 14) groups.

This retrospective cohort study included patients diagnosed with AK at Beijing Tongren Hospital between October 2015 and December 2024. The diagnosis of AK was based on typical clinical manifestations such as severe ocular pain, photophobia, and corneal infiltrates, in combination with positive microbiological findings. Laboratory investigations included corneal scrapings stained with Giemsa stain (Zhuhai Beisuo Biotechnology Co., Ltd., Zhuhai, China) and examined under light microscopy for the presence of trophozoites or cysts, as well as non-nutrient agar plate cultures overlaid with *Escherichia coli*, performed at the Ophthalmic Microbiology Laboratory of the Beijing Institute of Ophthalmology. A positive result from either test was considered diagnostic. Only patients with microbiologically confirmed AK were included in this study. Exclusion criteria included incomplete follow-up data and the presence of other forms of infectious keratitis. Two patients presented with bilateral AK. To maintain statistical independence and avoid inter-eye correlation, only one eye per patient (the first affected eye) was included in the final statistical analysis. Accordingly, 59 patients and 59 eyes were analyzed. A flowchart of patient selection is presented in Figure 1. The study was approved by the Institutional Review Board of Beijing Tongren Hospital (TRECKY2021-024), and all participants provided informed consent in line with the Declaration of Helsinki.

Throughout the study period (2015–2024), the *in vivo* confocal microscopy examinations were performed using the Rostock Cornea Module of the Heidelberg Retina Tomograph III (HRT III) (Heidelberg Engineering, Heidelberg, Germany), following a standardized imaging protocol that remained unchanged over time. All IVCM scans were conducted by trained corneal specialists using consistent acquisition settings and evaluation criteria. In addition, the core anti-*Acanthamoeba* treatment strategy, based on topical biguanides, remained consistent during the study period, with only minor adjustments in dosing frequency according to individual clinical response. Accordingly, methodological and therapeutic consistency was maintained throughout the study period.

### 2.2. Clinical Assessment and Prognostic Grouping

Baseline and clinical data were obtained from the electronic medical records and laboratory databases of Beijing Tongren Hospital. Demographic variables included sex, age, laterality, residence, and occupation. Risk factors comprised contact lens wear, ocular trauma, and exposure to contaminated water. Clinical evaluations included best-corrected visual acuity (BCVA) recorded in decimal units and corneal lesion–related signs, including epithelial defects, stromal infiltrates, ring infiltrates, groove-like stromal melting, and corneal neovascularization. AK severity was categorized into three stages: stage 1, infection confined to the corneal epithelium; stage 2, stromal infiltration without ring infiltrates; and stage 3, presence of ring infiltrates [1].

All patients received immediate anti-*Acanthamoeba* therapy with hourly 0.02% chlorhexidine and 0.02% polihexanide for the first week, tapered to four times daily from weeks 2 to 5. Maintenance treatment with 0.02% chlorhexidine monotherapy was given four times daily for 3 to 6 months, based on response. Patients with corneal perforation underwent therapeutic keratoplasty. Follow-up examinations, including visual acuity, slit-lamp photography, and IVCM, were performed at baseline, 1 month ± 7 days, and 3 months ± 7 days. Cases with poor outcomes were defined based on specific clinical criteria, including corneal perforation, the need for keratoplasty, or BCVA of 0.05 or worse (decimal notation). All remaining cases were classified as having a good outcome. This binary outcome definition was selected because these endpoints represent established indicators of treatment failure or severe visual impairment. Prognostic classification (poor vs. good) was retrospectively determined by two independent ophthalmologists under masked conditions, based on a comprehensive review of slit-lamp photographs, IVCM images, and clinical follow-up data. Discrepancies were resolved by a third senior corneal specialist.

### 2.3. In Vivo Confocal Microscopy Evaluation

IVCM was performed using the Heidelberg Retina Tomograph III with the Rostock Corneal Module. The device utilized a 670 nm diode laser to capture images at a resolution of 384 × 384 pixels within a 400 × 400 μm field of view. Before imaging, 0.5% proparacaine (Santen Pharmaceutical Co., Ltd., Osaka, Japan) was applied for topical anesthesia. Real-time eye positioning was guided using a built-in color CCD camera system. Scanning was independently performed by a trained corneal specialist in sequential slicing mode, with all clinical information masked. Scanning regions included the central cornea, four peripheral quadrants (superior, temporal, inferior, nasal), and the area encompassing the ulcer and adjacent tissue. Depth-resolved imaging was conducted from the epithelium to the endothelium, ultimately acquiring approximately 600 images per eye. All images were anonymized, coded by patient, and systematically evaluated for morphological features.

**Figure 2:**
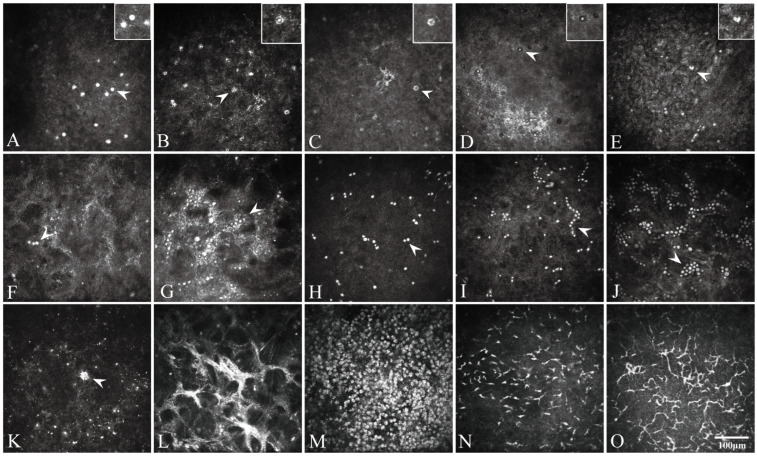
Representative *in vivo* confocal microscopy images showing characteristic morphological features observed in 59 patients with *Acanthamoeba* keratitis. (**A**) Bright spot cysts. (**B**) Double-walled cysts. (**C**) Signet ring cysts. (**D**) Cysts with target sign. (**E**) Coffee bean-shaped cysts. (**F**,**G**) Polygonal or stellate cysts. (**H**) Binary cysts. (**I**) Single chain of cysts. (**J**) Clustered cysts. (**K**) Trophozoite-like hyper-reflective structures. (**L**) Activated keratocytes. (**M**) Inflammatory cells. (**N**) Immature dendritic cells. (**O**) Mature dendritic cells with elongated projections. Scale bar: 100 μm. White arrows indicate the representative morphological features described in each panel.

Based on previous literature and our studies, a structured IVCM classification system for AK was established (Figure 2, Table 1). This system comprised three categories: (1) pathogen-related features (cysts and trophozoites). Cysts were further divided into six subtypes: bright spot, double-walled, signet ring, target-like, coffee bean-like, and stellate polygonal. (2) Cyst arrangement patterns: paired, linear (chain-like), and clustered formations. (3) Inflammatory response features: activated stromal cells, immature and mature dendritic cells, and other inflammatory cells. All features were assessed semi-quantitatively in binary form (presence = 1, absence = 0). Quantitative analysis included cyst counts per unit area and the measurement of maximum infiltration depth.

### 2.4. Feature-Outcome Association Analysis

To assess the association between IVCM features and clinical outcomes, 14 baseline morphological parameters were compared between prognosis groups. Qualitative variables (cyst morphology, arrangement, inflammatory response) were analyzed as binary variables (presence = 1, absence = 0), and quantitative variables (cyst density, maximal stromal depth) as continuous variables. Both types were further analyzed across prognostic subgroups and follow-up time points.

For prognostic modeling, baseline qualitative IVCM features were analyzed to avoid time-dependent bias. Variable selection followed two steps. First, univariable logistic regression identified candidate factors with *p* < 0.1. Second, considering the limited number of poor-prognosis cases (n = 14), the final multivariable model included a maximum of three predictors. Candidate variables were further assessed for collinearity (variance inflation factor < 2) and clinical relevance (including pathological plausibility and feasibility of detection in routine clinical practice) before inclusion in the final model. To address the limited number of poor-outcome cases (n = 14) and reduce small-sample bias, Firth’s penalized likelihood logistic regression was used for multivariable analysis.

### 2.5. Statistical Analysis

Continuous variables were tested for normality. Normally distributed variables were reported as mean ± standard deviation and compared using independent-sample *t*-tests. Non-normally distributed variables were reported as median (IQR) and compared using the Mann–Whitney U test. Categorical variables were summarized as counts and percentages and compared using Fisher’s exact test. Longitudinal changes in IVCM features were assessed using the Friedman test, and between-group comparisons (good vs. poor prognosis) of IVCM features, demographic characteristics, and clinical parameters were conducted using Fisher’s exact test or Mann–Whitney U test as appropriate.

For multivariable modeling and internal validation, Firth-corrected logistic regression was employed to identify independent predictors of poor prognosis, minimizing small-sample bias. Odds ratios (ORs) and 95% confidence intervals (CIs) were reported. Model performance was evaluated using receiver operating characteristic (ROC) curves (area under the curve [AUC], accuracy, sensitivity, and specificity), and internal validation was conducted via 5-fold cross-validation. Conventional logistic regression was conducted as a sensitivity analysis to confirm robustness, and variable importance was represented by |log(OR)|. All analyses were performed in R, with two-sided *p* < 0.05 considered statistically significant.

## 3. Results

### 3.1. Patient Characteristics and Clinical Manifestations

This study included 59 patients diagnosed with AK. The median age was 38.0 years (IQR: 17.5–54.5), and 32 patients (54.2%) were male. The left eye was more commonly affected (55.9%), and 59.3% of patients resided in rural areas. Common risk factors included contact lens use (37.3%), ocular trauma (32.2%), and exposure to tap water (27.1%). At baseline, 91.5% of patients had BCVA < 0.3. Clinically, 28 patients (47.4%) were classified as stage 2, and 25 (42.4%) as stage 3. Microbiological testing was positive in 81.3% of corneal scraping examinations and 67.3% of *Acanthamoeba* cultures (Table S1 and Figure S1).

The median follow-up duration was 110 days (IQR: 90.5–170.0). After 1 month of treatment, the proportion of patients with BCVA ≥ 0.3 increased from 8.5% to 13.6%. Clinical signs, including epithelial lesions, deep stromal infiltrates, and ring infiltrates, decreased modestly (57.6% to 52.5%, 47.4% to 42.3%, and 42.4% to 35.6%, respectively). Notably, the proportion of patients in stage 1 increased significantly to 23.7%, which was associated with a shift toward earlier disease stage. By the 3-months follow-up, further changes in clinical findings were observed: the proportion of patients with BCVA ≥ 0.3 more than tripled compared to baseline (27.1% vs. 8.5%). Clinical staging shifted markedly, with 50.7% of patients classified as stage 1 and only 17.0% remaining in stage 3. Neovascularization persisted in about one-third of patients throughout the follow-up. Finally, 45 patients (76.3%) achieved clinical remission and were classified as having a good prognosis, while 14 patients (23.7%) showed persistent or worsening symptoms and were classified as having a poor prognosis (Table 2).

### 3.2. Quantitative Analysis of IVCM Images

Fourteen representative qualitative IVCM features were analyzed and categorized into three groups: pathogen-related morphologies, cyst arrangement patterns, and inflammatory cellular responses (Table S2). At baseline, IVCM revealed bright spot cysts in 77.9% of cases, double-walled cysts in 37.2%, and target-like cysts in 50.8%. Trophozoites were detected in 24 cases (40.6%). Cysts arranged in cluster formations were observed in 49.1%, while activated keratocytes were present in 77.9%. Mature dendritic cells were identified in 61.0% of patients.

**Figure 3:**
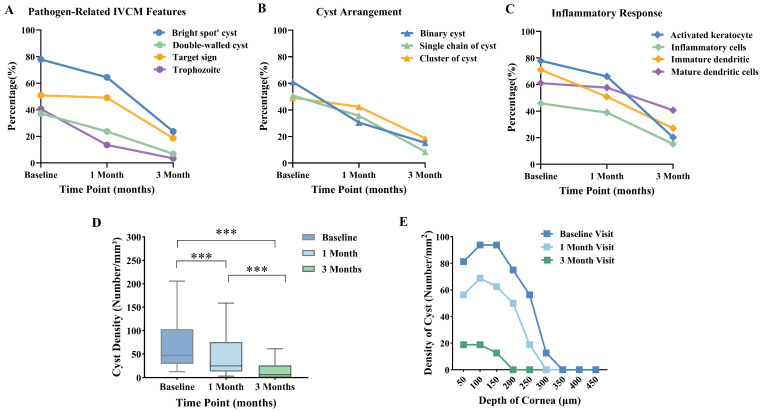
Analysis of IVCM morphological features at baseline, 1 month, and 3 months. (**A**) Pathogen-related features. (**B**) Cyst arrangement patterns. (**C**) Inflammatory response features. (**D**) Box plot showing cyst density (number/mm^2^) at three time points. (**E**) Density distribution along corneal depth layers. *** *p* < 0.001.

During follow-up, bright spot cysts demonstrated the greatest reduction, with detection rates decreasing from 77.9% at baseline to 64.4% at 1 month and 23.7% at 3 months. Double-walled cysts, target-like cysts, and trophozoites also declined markedly over time (Figure 3A). Clustered cysts exhibited a comparatively slower decline, from 49.1% to 18.6% (Figure 3B). Regarding inflammatory features, the proportion of activated keratocytes decreased from 77.9% to 20.3%, with parallel reductions in inflammatory cells and both immature and mature dendritic cells (Figure 3C).

Cyst density (number/mm^2^) declined significantly over time (*p* < 0.001), with median values decreasing from 46.88/mm^2^ at baseline to 25.00/mm^2^ at 1 month and 6.25/mm^2^ at 3 months (Figure 3D; Table S3). Spatially, cysts were mainly located in the mid-stromal region (150–250 μm) at baseline and decreased over follow-up. By 3 months, no cysts were detected in the deep stromal zone (≥350 μm), and overall density was markedly reduced (Figure 3E; Table S4).

### 3.3. Prognosis-Associated Differences in Baseline Features

Baseline demographic, clinical, and IVCM characteristics were compared between the good (n = 45) and poor prognosis (n = 14) groups (Table 3). In IVCM analysis, the poor prognosis group showed higher detection rates of double-walled cysts (64.2% vs. 28.8%, *p* = 0.017), trophozoites (64.2% vs. 33.3%, *p* = 0.039), and clustered cysts (78.5% vs. 40.0%, *p* = 0.015). Conversely, target-sign cysts (60.0% vs. 21.4%, *p* = 0.015), immature dendritic cells (77.7% vs. 50.0%, *p* = 0.045), and mature dendritic cells (73.3% vs. 21.4%, *p* = 0.001) were more frequently observed in the good prognosis group.

### 3.4. IVCM Features and Clinical Prognosis

**Figure 4:**
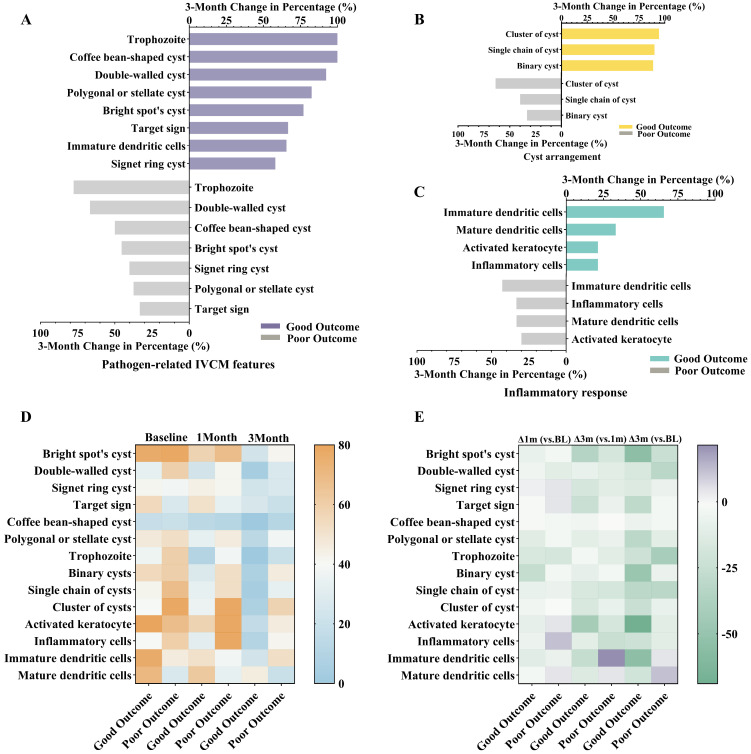
Longitudinal changes in qualitative IVCM features across prognosis groups. (**A**–**C**) Three-month percentage reductions in IVCM features, grouped by pathogen morphology, cyst arrangement, and inflammatory response. (**D**) Heatmap of feature detection rates at baseline, 1 month, and 3 months. (**E**) Heatmap of relative changes across timepoints (Δ1M vs. BL, Δ3M vs. 1M, Δ3M vs. BL) by prognosis group.

To assess prognosis-related longitudinal changes in IVCM features, the 3-month relative change in prevalence of 14 qualitative parameters was calculated, categorized into pathogen morphology, cyst arrangement, and inflammatory response (Figure 4 and Figure S2; Tables S5–S7). Overall, the good prognosis group exhibited consistently greater reductions across all domains compared to the poor prognosis group.

Among pathogen-related features (Figure 4A), trophozoites (100.0%), double-walled cysts (92.4%), and bright spot cysts (77.1%) showed marked reductions in the good prognosis group, whereas the poor prognosis group showed milder declines (77.7%, 40.1%, 45.4%, respectively). Regarding cyst arrangement (Figure 4B), the good prognosis group demonstrated marked reductions in binary (88.8%), single-chain (90.1%), and clustered cysts (94.5%), all notably greater than those in the poor group (33.3%, 40.0%, and 63.6%). These differences were associated with greater reductions in pathogen-related structures in eyes with favorable outcomes. For inflammatory features (Figure 4C), activated keratocytes and immature dendritic cells declined more markedly in the good prognosis group (83.3% and 65.6%, respectively) than in the poor prognosis group (30.0% and 42.8%).

Temporal heatmaps showed similar patterns. Figure 4D illustrates the raw detection rates of each feature at baseline, 1 month, and 3 months. Most features progressively declined in the good prognosis group, while the poor prognosis group exhibited delayed regression or persistent detection. To quantify the intensity of temporal changes, Figure 4E displays the relative change in detection rates across follow-up timepoints (Δ1 month vs. baseline, Δ3 months vs. 1 month, and Δ3 months vs. baseline). Consistent with the bar plot results, the good prognosis group showed steeper and more homogeneous declines, particularly in pathogen- and inflammation-related features.

Finally, to complement the qualitative findings, quantitative trends in cyst burden and stromal involvement depth were compared between the prognosis groups. The poor prognosis group consistently exhibited higher cyst density and deeper stromal infiltration throughout follow-up (Figure S3; Tables S8 and S9), showing higher cyst density and deeper stromal infiltration in association with unfavorable clinical outcomes.

### 3.5. Multivariable Regression Analysis of IVCM Features and Model Performance Evaluation

**Figure 5:**
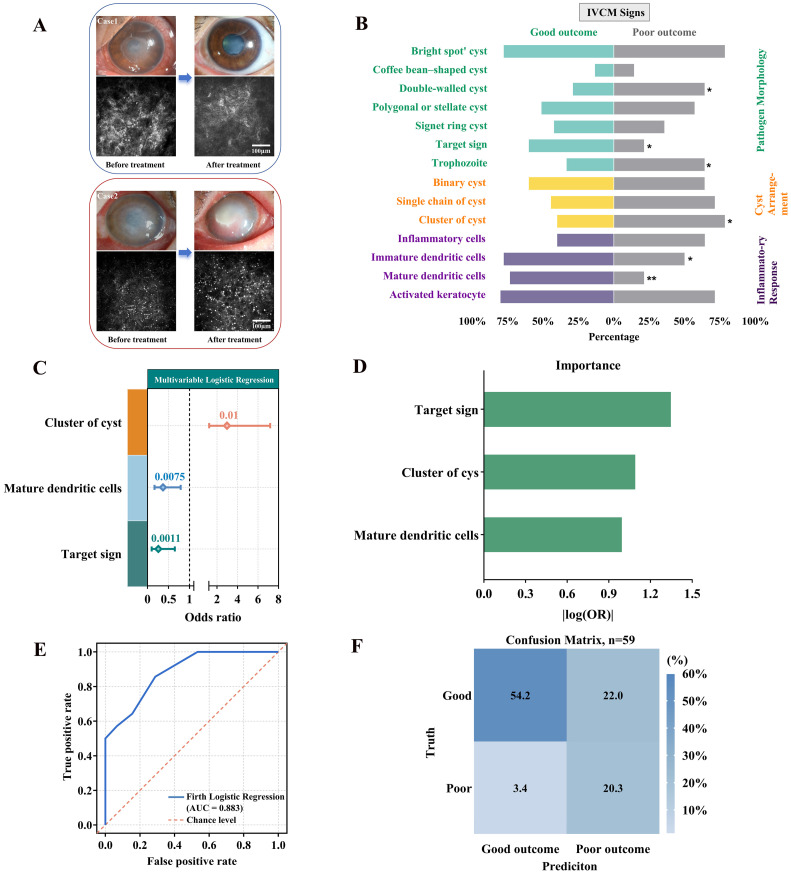
Prediction model based on baseline IVCM features and its performance validation. (**A**) Representative anterior segment and IVCM images before and after treatment for good and poor prognosis groups. (**B**) Baseline detection rates of IVCM features, with statistical significance indicated by asterisks: * *p* < 0.05, ** *p* < 0.01. (**C**) Firth-corrected logistic regression model using three IVCM features. Clustered cysts were identified as a risk factor for poor prognosis, while target-like cysts and mature dendritic cells were protective. (**D**) Relative contribution of each variable to the predictive model. (**E**) Receiver operating characteristic (ROC) curve showing good model performance (AUC = 0.883). (**F**) Confusion matrix showing classification results. The model correctly identified 12 of 14 patients with poor prognosis (sensitivity 85.7%) and achieved an overall accuracy of 74.6%.

To further assess the prognostic value of baseline IVCM features, cases with different clinical outcomes were reviewed. As illustrated in Figure 5A, the good-prognosis case showed a rapid reduction in cyst detectability and improvement in stromal appearance, whereas the poor-prognosis case exhibited persistent cysts and ongoing stromal abnormalities. These observations prompted quantitative analysis using logistic regression.

Univariable logistic regression was performed on 14 candidate features (Figure 5B, Table S10), followed by multivariable modeling using Firth-corrected logistic regression. In this model, clustered cysts were independently associated with poor prognosis (OR = 2.98, 95% CI: 1.24–7.19, *p* = 0.010), whereas target sign cysts (OR = 0.26, 95% CI: 0.10–0.65, *p* = 0.001) and mature dendritic cells (OR = 0.37, 95% CI: 0.17–0.79, *p* = 0.008) were inversely associated with poor prognosis (Table 4; Figure 5C). Notably, target sign cysts showed the highest relative importance in the model, as indicated by variable importance analysis (Figure 5D). The model demonstrated good discriminative performance, with an AUC of 0.883 (Figure 5E), achieving 74.6% accuracy, 85.7% sensitivity, and 71.1% specificity in identifying poor-prognosis cases (Figure 5F). Robustness was supported by consistent findings in sensitivity analysis using conventional multivariable logistic regression. Clustered cysts remained a significant risk factor (OR = 3.54, *p* = 0.014), while target sign cysts (OR = 0.21, *p* = 0.004) and mature dendritic cells (OR = 0.31, *p* = 0.011) continued to show protective associations (Table S11).

## 4. Discussion

This study presents a comprehensive longitudinal evaluation of IVCM features in *Acanthamoeba* keratitis, capturing temporal changes in pathogen density, spatial arrangement, and host immune response throughout treatment. In contrast to previous studies that primarily focused on diagnostic imaging, our findings provide a broader perspective on disease progression and its prognostic implications [14,21,22,23,24]. We systematically monitored these features across three predefined treatment stages and identified baseline indicators independently associated with clinical outcomes. These variables were incorporated into a Firth-corrected logistic regression model, which demonstrated good discriminative performance. Overall, our findings may expand the clinical role of IVCM from static imaging to dynamic monitoring, providing a quantitative framework to support early risk stratification and individualized management of AK [25,26,27,28].

Longitudinal analysis of cyst morphologies revealed distinct clearance and recovery patterns between prognosis groups. Our results extend previous cross-sectional studies by demonstrating dynamic morphological changes over time [13]. In the good-prognosis group, target-like cysts declined rapidly, nearly disappearing by 3 months, which may be associated with greater treatment responsiveness and potentially reflect transitional or labile pathogen forms, as suggested by Li et al. [29]. In contrast, paired and clustered cysts were associated with poor outcomes, with residual detection at 3 months, consistent with Chopra et al.’s report of higher clustered cyst prevalence in severe AK [12]. Their compact structure may be associated with enhanced survival, deeper stromal localization, or immune evasion. These patterns highlight the biological heterogeneity of *Acanthamoeba* infection and suggest that certain IVCM features may have potential prognostic relevance during treatment [30]. Unlike prior studies emphasizing morphological diagnosis, this study first characterized the temporal evolution of cyst types and their association with clinical outcomes.

We identified cyst density and stromal depth, in addition to morphological features, as variables independently associated with clinical outcomes. Patients with favorable outcomes showed a marked decline in cyst density and superficial distribution, whereas poor-outcome cases maintained higher densities and deeper stromal involvement, particularly clustered cysts beyond 350 μm. These findings are consistent with prior IVCM studies linking persistent or deeply embedded cysts to prolonged disease or treatment resistance [12,15]. We propose a “density–depth–distribution” triad as a novel IVCM-based prognostic axis in AK. Prior studies have shown that *Acanthamoeba* cysts may penetrate deeper layers through active motility or chemotactic cues, where reduced drug penetration and immune evasion may impair clearance [31,32]. Thus, IVCM may offer value beyond diagnosis by enabling longitudinal assessment of infection-related changes and tissue integrity.

Our results also demonstrated that deeper stromal involvement was more prevalent in patients with poor outcomes, particularly when clustered or paired cysts were located beyond 250–350 μm. This finding suggests that infection persisting in deeper corneal layers may be associated with reduced treatment effectiveness [33,34,35,36]. The compact lamellar organization and limited vascularity of the posterior stroma hinder drug penetration and immune surveillance, potentially allowing for *Acanthamoeba* to evade host clearance mechanisms [37,38]. Previous reports have similarly associated deep-seated cysts with prolonged infection and poor therapeutic response [39,40]. Moreover, clustered cysts in deep layers may indicate chronic infection niches with high stress resistance, which may warrant closer monitoring or consideration of intensified treatment strategies [40,41,42]. These observations underscore the importance of early identification and targeted intervention before the pathogen infiltrates deeper corneal layers.

To enhance clinical applicability, a prognostic model was developed using baseline IVCM features. Firth-corrected logistic regression was applied to ensure robust estimation with a limited sample size, as this penalized likelihood method reduces bias in small or imbalanced datasets [43]. The model incorporated biologically meaningful features. Clustered cysts, previously associated with unfavorable clinical status [12,30], may reflect dense pathogen aggregations. Target-like cysts, considered fragile or transitional forms [29], declined more rapidly in cases with favorable outcomes. The presence of mature dendritic cells, indicative of an active immune response in ocular infection contexts, may be associated with more effective host–pathogen interactions. Mature dendritic cells, reflecting active immune surveillance, were more frequent in good-prognosis eyes at baseline and declined over time, consistent with a more regulated immune response [29]. Conversely, persistently elevated inflammatory cells were more frequently observed in poor-outcome eyes, consistent with prolonged inflammatory activity.

This divergence in host response underscores the value of IVCM in capturing both pathogen morphology and host–pathogen interactions. Similar observations have reported activated immune cells in infectious keratitis [17], yet no prior studies have correlated temporal changes in dendritic and inflammatory cells with clinical outcomes in AK. The model achieved good sensitivity (85.7%) and accuracy (74.6%) in identifying poor prognosis. Although previous studies have reported associations between individual IVCM features, including cyst density and immune cell infiltration, and clinical outcomes in AK [18,21,44], a standardized and validated prognostic framework incorporating multiple IVCM parameters has not been fully developed. By combining quantitative information on pathogen morphology, spatial distribution, and host immune response across treatment stages, our study provides a structured approach for prognostic risk stratification based on IVCM imaging.

In clinical practice, follow-up schedules in AK are often inconsistent due to the disease’s heterogeneous course. However, as a national referral center for corneal diseases, our institution follows standardized protocols, with IVCM performed by the same imaging team and integrated into routine outpatient care. Although the recruitment period spanned nearly a decade (2015–2024), imaging protocols, device models, and core anti-Acanthamoeba treatment strategies remained consistent throughout the study period, reducing potential temporal heterogeneity related to evolving clinical practice. Only patients with complete IVCM data at predefined intervals were included, which limited the sample size but ensured imaging quality, temporal comparability, and meaningful dynamic evaluation. The study aimed to predict long-term prognosis based on early follow-up data, focusing on baseline to 1-month and 3-month changes. This time window represents the most active treatment phase in AK, when key shifts in cyst burden, inflammatory response, and structural recovery often occur. These early imaging dynamics were closely associated with clinical outcomes and may provide useful insights for follow-up assessment.

Despite the retrospective design and limited follow-up duration, the model demonstrates good reproducibility and clinical applicability. It offers a structured framework for IVCM-based risk assessment and highlights the importance of early-stage imaging dynamics. Future research should pursue multicenter validation, extend the follow-up period, and explore the integration of IVCM findings with clinical indicators and automated image analysis to improve the precision and scalability of prognostic modeling in AK.

## 5. Conclusions

This study demonstrates that IVCM enables the quantitative assessment of prognostic features in AK by capturing dynamic changes in pathogen morphology, stromal distribution, and immune response. A Firth-corrected logistic regression model based on 14 systematically assessed IVCM features showed good prognostic discriminative ability. Higher cyst burden, clustered cysts, and persistent stromal involvement indicated poorer prognosis, whereas target-like cysts and mature dendritic cells indicated better prognosis. These findings highlight the potential of IVCM not only as a diagnostic method but also as a practical tool for early outcome prediction and individualized clinical management. Further prospective, multicenter studies are warranted to validate and generalize this imaging-based prognostic framework.

## Figures and Tables

**Table 1:** Morphological classification of IVCM features for *Acanthamoeba* keratitis.

Classification	Morphological Description	Size (μm)	Reference
Cyst			
Bright spot	Round, oval, hyper-reflective particles or refractile bodies typically measure	10–30	[3,6,10]
Double wall	Round structures with a bi-layered or double walled hyper-reflective appearance	10–30	[20]
Signet ring	Hyper-reflective wall or outer ring with a dark center or less reflectivity inside the ring	5–20	[3,20]
Target sign	Low-refractile outer wall with a halo and a bright center or hyper-reflective nucleus	5–20	[6]
Coffee bean-shaped	Paired cyst reflections resembling kidney or coffee beans, usually measuring	5–20	[5,10]
Polygonal or stellate	Polygonal or stellate hyper-reflective objects	10–30	[7]
Binary cysts	Paired or closely adjacent round or ovoid hyper-reflective structures	5–20	[20]
Single chain	Linear or single-file arrangement of round or ovoid hyper-reflective objects	5–20	[20]
Cluster of cysts	Clusters of round or ovoid hyper-reflective objects	5–20	[20]
**Trophozoite**	Irregular, pear or wedge shaped hyper-reflective structures with acanthopodia or pseudopods	20–100	[3,20]
**Inflammatory cell**			
Immature DCs	Round or oval shape with fewer and shorter projections	10–15	[5,20]
Mature DCs	Bright specular structures with dendriform processes	15–55	[5,20]
Others	Irregular, oval-shaped hyper-reflective structures, lacking dendritic processes or external walls, resembling leukocytes or neutrophils	10–40	[6,20]
**Activated keratocyte**	High reflectivity arranged in a honeycomb pattern	50–75	[5,20]

**Table 2:** Clinical and microbiological examinations of 59 patients with *Acanthamoeba* keratitis.

Parameters	Baseline	1 Month	3 Months
Visual acuity, n (%)			
<0.05	14 (23.7)	14 (23.7)	13 (22.0)
0.05~0.1	12 (20.3)	5 (8.5)	3 (5.1)
0.1~0.3	28 (47.5)	32 (54.2)	27 (45.8)
≥0.3	5 (8.5)	8 (13.6)	16 (27.1)
**Clinical signs, n (%)**			
Epithelial lesions	34 (57.6)	31 (52.5)	32 (54.2)
Deep stromal infiltrates	28 (47.4)	25 (42.3)	15 (25.4)
Ring infiltrates	25 (42.4)	21 (35.6)	10 (16.9)
Groove-shaped melting	15 (25.4)	15 (25.4)	9 (15.2)
Neovascularization	21 (35.6)	21 (35.6)	18 (30.5)
**Clinical stage, n (%)**			
Stage 1	6 (10.2)	14 (23.7)	30 (50.7)
Stage 2	28 (47.4)	24 (40.7)	19 (32.3)
Stage 3	25 (42.4)	21 (35.6)	10 (17.0)

**Table 3:** Comparison of baseline demographic, clinical, and IVCM features between patients with good and poor outcomes in *Acanthamoeba* keratitis.

	Good Outcome	Poor Outcome	*p*-Value
**Age [Median (IQR), years]**	27.0 (17.0–50.0)	58.5 (42.3–63.8)	0.0008
**Gender, n (%)**			0.803
Male	24 (53.3)	8 (57.1)	
Female	21 (46.7)	6 (42.9)	
**IVCM morphological signs, n (%)**			
**Pathogens**			
Bright spot cyst	35 (77.7)	11 (78.5)	1.000
Double-walled cyst	13 (28.8)	9 (64.2)	0.017
Signet ring cyst	19 (42.2)	5 (35.7)	0.665
Target sign	27 (60.0)	3 (21.4)	0.015
Coffee bean–shaped cyst	6 (13.3)	2 (14.3)	1.000
Polygonal or stellate cyst	23 (51.1)	8 (57.1)	0.693
Trophozoite	15 (33.3)	9 (64.2)	0.039
**Cyst Arrangement, n (%)**			
Binary cyst	27 (60.0)	9 (64.2)	0.774
Single chain of cysts	20 (44.4)	10 (71.4)	0.125
Cluster of cysts	18 (40.0)	11 (78.5)	0.015
**Inflammatory Response, n (%)**			
Activated keratocyte	36 (80.0)	10 (71.4)	0.485
Inflammatory cells	18 (40.0)	9 (64.3)	0.314
Immature dendritic cells	35 (77.7)	7 (50.0)	0.045
Mature dendritic cells	33 (73.3)	3 (21.4)	0.001

**Table 4:** Multivariable logistic regression of baseline IVCM features for clinical prognosis.

Variable	Logistic Coefficient	Odds Ratio (95% CI)	*p*-Value
Target sign	−1.3456	0.26 (0.10–0.65)	0.0011
Cluster of cysts	1.0934	2.98 (1.24–7.19)	0.0102
Mature dendritic cells	−1.0035	0.37 (0.17–0.79)	0.0075

## Data Availability

The data that support the findings of this study are available from the corresponding author upon reasonable request.

## Supplementary Materials

The following supporting information can be downloaded at https://www.mdpi.com/article/10.3390/pathogens15030262/s1. Figure S1: Baseline distribution of demographic, clinical, and IVCM features in patients with *Acanthamoeba* keratitis; Figure S2: Comparison of cyst density and stromal depth distribution between prognostic groups during follow-up. Table S1: Demographic, clinical, and *in vivo* confocal microscopy features of the 59 patients with *Acanthamoeba* keratitis; Table S2: Distribution of 14 IVCM morphological indicators across baseline, 1-month, and 3-month visits; Table S3: Cyst density (cysts/mm^2^) at baseline, 1-month, and 3-month visits; Table S4: Cyst density (number/mm^2^) across corneal depth at different follow-up time points; Table S5: Temporal prevalence of IVCM features in different prognostic groups at baseline, 1-month, and 3-month follow-up; Table S6: Three-month changes in IVCM feature prevalence between prognostic groups; Table S7: Relative and absolute changes in IVCM feature prevalence at 3-month follow-up in different prognosis groups; Table S8: Cyst density (number/mm^2^) by corneal depth and prognosis group across follow-up; Table S9: Prevalence of different cyst arrangements by stromal depth and prognostic outcome; Table S10: Baseline IVCM features included in the analysis; Table S11: Multivariable logistic regression results using the conventional method.

## Abbreviations

The following abbreviations are used in this manuscript:

AK	*Acanthamoeba* keratitis
IVCM	*In vivo* confocal microscopy
BCVA	Best-corrected visual acuity
DCs	Dendritic cells
