# Supplementary material for: In Vivo Confocal Microscopy as a Prognostic Indicator in Acanthamoeba Keratitis: Insights from a Retrospective Study
Source: Pathogens. 2026 Mar 2;15(3):262. doi: 10.3390/pathogens15030262 (PMC13028759; doi:10.3390/pathogens15030262)
Supplement: Supplementary file 1 [file pathogens-15-00262-s001.zip › pathogens-4146723-supplementary.pdf]

***In Vivo* Confocal Microscopy as a Prognostic Indicator in *Acanthamoeba* Keratitis: Insights from a Retrospective Study**

Supplementary Materials

Figure S1. Baseline distribution of demographic, clinical, and IVCM features in patients with *Acanthamoeba* keratitis

Figure S2. Comparison of cyst density and stromal depth distribution between prognostic groups during follow-up

Table S1. Demographic, clinical, and *in vivo* confocal microscopy features of the 59 patients with *Acanthamoeba* keratitis

Table S2. Distribution of 14 IVCM morphological indicators across baseline, 1-month, and 3-months visits

Table S3. Cyst density (cysts/mm^2) at baseline, 1-month, and 3-months visits

Table S4. Cyst density (number/mm^2) across corneal depth at different follow-up timepoints

Table S5. Temporal prevalence of IVCM features in different prognostic groups at baseline, 1-month, and 3-months follow-up

Table S6. Three-month changes in IVCM feature prevalence between prognostic groups

Table S7. Relative and absolute changes in IVCM feature prevalence at 3-month follow-up in different prognosis groups

Table S8. Cyst density (number/mm^2) by corneal depth and prognosis group across follow-up

Table S9. Prevalence of different cyst arrangements by stromal depth and prognostic outcome

Table S10. All variables represent baseline IVCM features

Table S11. Multivariable logistic regression results using the conventional method

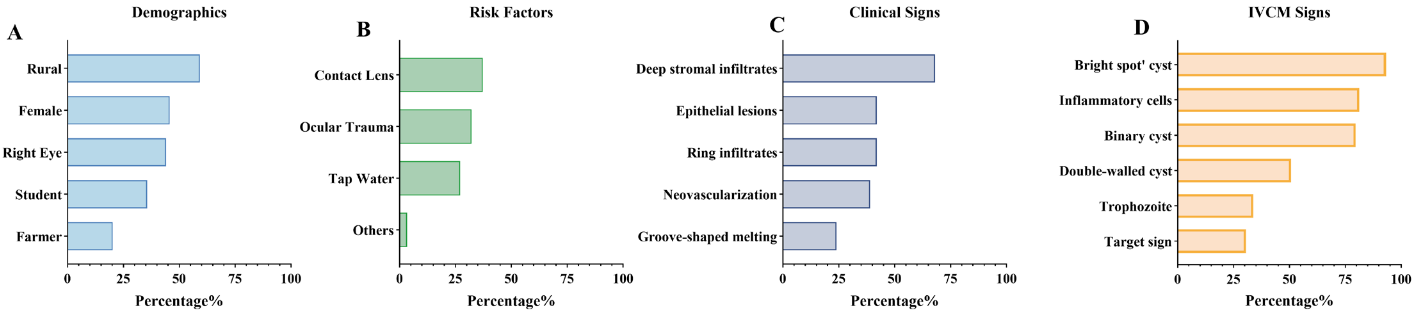

Supplementary Figure S1. Baseline distribution of demographic, clinical, and IVCM features in patients with *Acanthamoeba keratitis*. Bar charts display the frequency distributions of (A) demographic characteristics, (B) risk factors, (C) clinical signs at presentation, and (D) IVCM indicators in the study cohort (n = 59).

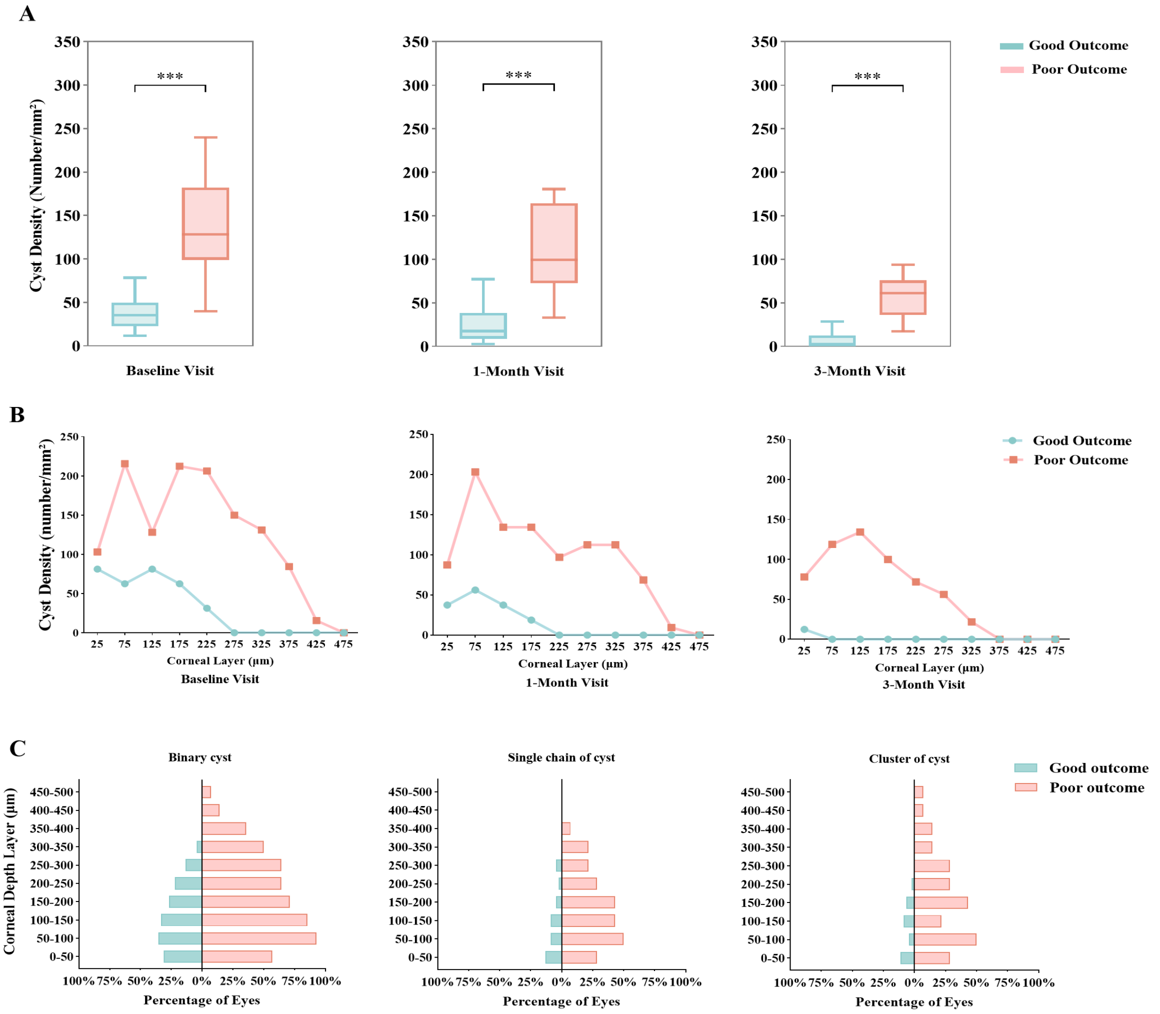

Supplementary Figure S2. Comparison of cyst density and stromal depth distribution between prognostic groups during follow-up. (A) Cyst density per unit area at baseline, 1 month, and 3 months in the good vs. poor prognosis groups. The poor prognosis group exhibited consistently higher cyst density across all time points. (B) Distribution of cyst density across different stromal depth layers. The poor prognosis group showed greater density and deeper stromal involvement. (C) Depth distribution of three cyst arrangement patterns. In the poor prognosis group, paired, linear, and clustered cysts more frequently extended into mid-to-posterior stromal layers (⩾250μm), exhibiting deeper and denser involvement.

Supplementary Table S1. Demographic, clinical, and *in vivo* confocal microscopy features of the 59 patients with *Acanthamoeba* keratitis.

Parameters	Results
Age, years [Median (IQR)]	38.0 (17.5-54.5)
Gender [n (%)]	
Male	32 (54.2)
Female	27 (45.8)
Eye [n (%)]	
Right	26 (44.1)
Left	33 (55.9)
Region [n (%)]	
Rural	35 (59.3)
Urban	24 (40.7)
Occupation [n (%)]	
Student	22 (37.3)
Farmer	9 (15.3)
Worker	20 (33.9)
Others	8 (13.5)
Risk factors [n (%)]	
Contact Lens	22 (37.3)
Ocular Trauma	19 (32.2)
Tap Water	16 (27.1)
Others	2 (3.4)
Topical steroid use	36 (61.0)
Visual acuity [n (%)]	
<0.05	14 (23.7)
0.05~0.1	12 (20.3)
0.1~0.3	28 (47.5)
⩾0.3	5 (8.5)
Clinic signs [n (%)]	
Epithelial lesions	25 (42.3)
Deep stromal infiltrates	28 (47.4)
Ring infiltrates	25 (42.3)
Groove-shaped melting	15 (25.4)
Neovascularization	21 (35.6)
Clinical stage [n (%)]	
Stage 1	6 (10.2)
Stage 2	28 (47.4)
Stage 3	25 (42.4)
Positive microbiological examination [n (%)]	
Scraping	48 (81.3)
Culture	35 (67.3)
IVCM signs [n (%)]	
Bright spot' cyst	46 (77.9)

Double-walled cyst	22 (37.2)
Signet ring cyst	24 (40.6)
Target sign	30 (50.8)
Coffee bean–shaped cyst	8 (13.5)
Polygonal or stellate cyst	31 (52.5)
Trophozoite	24 (40.6)
Binary cyst	36 (61.0)
Single chain of cysts	30 (50.8)
Cluster of cysts	29 (49.1)
Activated keratocyte	46 (77.9)
Inflammatory cells	27 (45.8)
Immature dendritic cells	42 (71.1)
Mature dendritic cells	36 (61.0)
Duration, days [Median (IQR)]	110 (90.5-170.0)
Prognosis [n (%)]	
Good outcome	45（76.3）
Poor outcome	14（23.7）

Supplementary Table S2. Distribution of 14 IVCM morphological indicators across baseline, 1-month, and 3-months visits.

IVCM morphological signs	Baseline	1 Month	3 Months
Bright spot' cyst	46 (77.9%)	38 (64.4%)	14 (23.7%)
Double-walled cyst	22 (37.2%)	14 (23.7%)	4 (6.8%)
Signet ring cyst	24 (40.6%)	27 (45.8%)	11 (18.6%)
Target sign	30 (50.8%)	29(49.1%)	11 (18.6%)
Coffee bean-shaped cyst	8 (13.5%)	5 (8.5%)	1 (1.6%)
Polygonal or stellate cyst	31 (52.5%)	20 (33.9%)	9(15.3%)
Trophozoite	24 (40.6%)	8 (13.5%)	2 (3.4%)
Binary cyst	36 (61.0%)	18(30.5)	9 (15.3%)
Single chain of cyst	30 (50.8%)	21(35.6)	5(8.5)
Cluster of cysts	29 (49.1%)	25(42.4)	11(18.6)
Activated keratocyte	46(77.9%)	39(66.1%)	12(20.3)
Inflammatory cells	27(45.8%)	23(38.9%)	9(15.3)
Immature dendritic cells	42 (71.1%)	30(50.8%)	16(27.1%)
Mature dendritic cells	36 (61.0%)	34(57.6%)	24(40.7%)

Supplementary Table S3. Cyst density (cysts/mm²) at baseline, 1-month, and 3-months visits.

**Visit**	**Median Density (cysts/mm²)**	**IQR (cysts/mm²)**
Baseline	46.88	29.69-94.69
1 Month	25.00	13.44-71.25
3 Month	6.25	0.00-25.94

Supplementary Table S4. Cyst density (number/mm^2) across corneal depth at different follow-up timepoints.

Corneal Depth (μm)	Baseline	1 Month	3 Months
0-50	81.25	56.25	18.75
50-100	93.75	68.75	18.75
100-150	93.75	62.50	12.50
150-200	75.00	50.00	0.00
200-250	56.25	18.75	0.00
250-300	12.50	0.00	0.00
300-350	0.00	0.00	0.00
350-400	0.00	0.00	0.00
400-450	0.00	0.00	0.00

Supplementary Table S5. Temporal prevalence of IVCM features in different prognostic groups at baseline, 1-month, and 3-months follow-up.

IVCM Feature	Baseline Outcome Group (%)		1-Month Outcome Group (%)		3-Months Outcome Group (%)	
	Good (n=45)	Poor (n=14)	Good (n=45)	Poor (n=14)	Good (n=45)	Poor (n=14)
Bright spot's cyst	35 (77.7)	11 (78.5)	28(62.2)	10(71.4)	8(17.8)	6(42.9)
Double-walled cyst	13 (28.8)	9 (64.2)	8(17.8)	6(42.8)	1(2.2)	3(21.4)
Signet ring cyst	19 (42.2)	5 (35.7)	21(46.6)	6 (42.8)	8(17.7)	3 (21.4)
Target sign	27 (60.0)	3 (21.4)	25(55.6)	4(28.5)	9(20.0)	2(14.3)
Coffee bean-shaped cyst	6 (13.3)	2 (14.3)	4(8.9)	1(7.1)	0(0.0)	1(7.1)
Polygonal or stellate cyst	23 (51.1)	8 (57.1)	13(28.9)	7(50.0)	4(8.9)	5(35.7)
Trophozoite	15 (33.3)	9 (64.2)	3(6.7)	5(35.7)	0(0.0)	2 (14.3)
Binary cysts	27 (60.0)	9 (64.2)	14(31.1)	8(57.1)	3(6.7)	6(42.8)
Single chain of cysts	20 (44.4)	10 (71.4)	5(11.1)	7(50.0)	2(4.4)	6(42.8)
Cluster of cysts	18 (40.0)	11 (78.5)	7(15.6)	6(42.9)	1(2.2)	4(28.6)
Activated keratocyte	36 (80.0)	10 (71.4)	28(62.2)	11(78.5)	6(13.3)	7(50.0)
Inflammatory cells	18(40.0)	9(64.3)	12(26.7)	11(78.6)	5(11.1)	6(42.9)
Immature dendritic cells	35 (77.7)	7 (50.0)	25(55.6)	5(35.7)	12(26.7)	4(28.6)
Mature dendritic cells	33 (73.3)	3 (21.4)	30(66.7)	4(28.6)	22(48.9)	2(14.3)

Supplementary Table S6. Three-month changes in IVCM feature prevalence between prognostic groups.

IVCM Feature	Δ1m vs Baseline (Good)	Δ1m vs Baseline (Poor)	Δ3m vs 1m (Good)	Δ3m vs 1m (Poor)	Δ3m vs Baseline (Good)	Δ3m vs Baseline (Poor)
Bright spot's cyst	-15.5	-7.1	-44.4	-28.5	-59.9	-35.6
Double-walled cyst	-11.0	-21.4	-15.6	-21.4	-26.6	-42.8
Signet ring cyst	4.4	7.1	-28.9	-21.4	-24.5	-14.3
Target sign	-4.4	7.1	-35.6	-14.2	-40.0	-7.1
Coffee bean-shaped cyst	-4.4	-7.2	-8.9	0	-13.3	-7.2
Polygonal or stellate cyst	-22.2	-7.1	-20	-14.3	-42.2	-21.4
Trophozoite	-26.6	-28.5	-6.7	-21.4	-33.3	-49.9
Binary cyst	-37.8	-7.1	-17.8	-7.1	-53.3	-21.4
Single chain of cyst	-15.5	-14.3	-26.7	-28.5	-40.0	-28.6
Cluster of cyst	-8.9	0.1	-26.7	-16.3	-37.8.	-49.9
Activated keratocyte	-17.8	7.1	-51.1	-28.5	-66.7	-21.4
Inflammatory cells	-13.3	14.3	-20	-35.7	-28.9	-21.4
Immature dendritic cells	-22.1	-14.3	-37.8	21.4	-51.0	-21.4
Mature dendritic cells	-6.6	7.2	-17.8	-14.3	-24.4	-7.1

Supplementary Table S7. Relative and absolute changes in IVCM feature prevalence at 3-month follow-up in different prognosis groups.

IVCM Feature	Δ3m vs BL (Good)	Good BL	Relative Δ3m vs BL (Good)	Δ3m vs BL (Poor)	Poor BL	Relative Δ3m vs BL (Poor)
Bright spot's cyst	-59.9	77.7	-77.1	-35.6	78.5	-45.4
Double-walled cyst	-26.6	28.8	-92.4	-42.8	64.2	-66.7
Signet ring cyst	-24.5	42.2	-58.1	-14.3	35.7	-40.1
Target sign	-40.0	60.0	-66.7	-7.1	21.4	-33.2
Coffee bean-shaped cyst	-13.3	13.3	-100	-7.2	14.3	-50.0
Polygonal or stellate cyst	-42.2	51.1	-82.6	-21.4	57.1	-37.4
Trophozoite	-33.3	33.3	-100	-49.9	64.2	-77.7
Binary cyst	-53.3	60.0	-88.8	-21.4	64.2	-33.3
Single chain of cyst	-40.0	44.4	-90.1	-28.6	71.4	-40.0
Cluster of cyst	-37.8.	40.0	-94.5	-49.9	78.5	-63.6
Activated keratocyte	-66.7	80.0	-83.3	-21.4	71.4	-30.0
Inflammatory cells	-28.9	40.0	-72.2	-21.4	64.3	-33.3
Immature dendritic cells	-51.0	77.7	-65.6	-21.4	50.0	-42. 8
Mature dendritic cells	-24.4	73.3	-33.3	-7.1	21.4	-33.2

Supplementary Table S8. Cyst density (number/mm²) by corneal depth and prognosis group across follow-up.

Corneal Depth (μm)	Baselin			1-Month			3-Month		
	Good	Poor	*P*-Value	Good	Poor	*P*-Value	Good	Poor	*P*-Value
0-50	81.25	103.13	0.114	37.5	87.50	0.003	12.5	78.13	0.00003
50-100	62.50	215.63	0.00	56.25	203.13	0.00007	0.00	118.75	0.00
100-150	81.25	128.13	0.008	37.5	134.378	0.00001	0.00	134.375	0.00
150-200	62.50	212.50	0.00001	18.75	134.38	0.00004	0.00	100.00	0.00
200-250	31.25	206.25	0.00	0.00	96.88	0.00	0.00	71.88	0.00
250-300	0.00	150.00	0.00	0.00	112.50	0.00	0.00	56.25	0.00
300-350	0.00	131.25	0.00	0.00	112.50	0.00	0.00	21.88	0.00
350-400	0.00	84.38	0.00	0.00	68.75	0.00	0.00	0.00	0.00004
400-450	0.00	15.63	0.00	0.00	9.38	0.00	0.00	0.00	0.002
450-500	0.00	0.00	0.00004	0.00	0.00	0.00004	0.00	0.00	0.08

Note: Cyst densities are listed for 10 stratified corneal layers (0-500 μm, in 50 μm increments) at each time point. Comparisons between good and poor outcome groups were conducted using appropriate non-parametric tests. P-values indicate significant differences in cyst infiltration depth.

Supplementary Table S9. Prevalence of different cyst arrangements by stromal depth and prognostic outcome.

Corneal Depth	Binary cyst		Single chain of cyst		Cluster of cysts	
Layer(μm)	Good (%)	Poor (%)	Good (%)	Poor (%)	Good (%)	Poor (%)
0-50	31.11	57.14	13.33	28.58	11.11	28.58
50-100	35.56	92.86	8.89	50.00	4.44	50.00
100-150	33.33	85.71	8.89	42.86	8.89	21.43
150-200	26.67	71.43	4.44	42.86	6.67	42.86
200-250	22.22	64.29	2.22	28.57	2.22	28.57
250-300	13.33	64.29	4.44	21.42	0.00	28.57
300-350	4.44	50.00	0.00	21.43	0.00	14.29
350-400	0.00	35.71	0.00	7.143	0.00	14.29
400-450	0.00	14.29	0.00	0.00	0.00	7.14
450-500	0.00	7.14	0.00	0.00	0.00	7.14

Note: This table shows the percentage of eyes with detectable binary, single-chain, and clustered cysts across 10 corneal layers (0–500 μm) in the good and poor outcome groups. The data emphasize the deeper localization of complex cyst forms in poor-prognosis cases.

Supplementary Table S10. All variables represent baseline IVCM features. Odds Ratios (OR) and 95% Confidence Intervals (CI) are reported based on univariate logistic regression.

**Variable**	**OR**	**95% CI**	***P*-value**
Age (years)	1.06	1.02–1.11	0.004
Double-walled cyst	4.43	1.29–16.92	0.022
Bright spot cyst	1.05	0.26–5.28	0.950
Signet ring cyst	0.76	0.21–2.58	0.666
Target sign	0.18	0.04–0.68	0.018
Coffee bean–shaped cyst	1.08	0.15–5.46	0.928
Polygonal or stellate cyst	1.28	0.38–4.44	0.693
Trophozoite	3.60	1.06–13.56	0.046
Binary cyst	1.20	0.35–4.45	0.774
Single chain of cyst	3.12	0.90–12.79	0.086
Cluster of cysts	5.50	1.48–26.90	0.018
Activated keratocyte	0.63	0.15–2.69	0.506
Inflammatory cells	2.70	0.80–10.05	0.118
Immature dendritic cells	0.29	0.08–1.01	0.052
Mature dendritic cells	0.10	0.02–0.38	0.002

Supplementary Table S11. Multivariable logistic regression results using the conventional method. The table presents the odds ratio (OR), 95% confidence interval (CI), and P-value for each selected IVCM feature included in the final model to verify the consistency of multivariable associations in sensitivity analysis.

**Variable**	**OR**	**95% CI**	***P*-value**
Cluster of cysts	3.54	1.40–11.18	0.0141
Target sign	0.21	0.06–0.54	0.0038
Mature dendritic cells	0.31	0.11–0.71	0.0110
